# Thyroid hormone resistance syndrome with P453T mutation in thyroid hormone receptor β gene: A pedigree report

**DOI:** 10.1097/MD.0000000000022824

**Published:** 2020-10-30

**Authors:** Ayiguli Yusufu, Wen-Jing Chen, Ming-Chen Zhang

**Affiliations:** a Department of Endocrinology, Xinjiang Medical University affiliated First Hospital; b School of Public Health, Xinjiang Medical University, Urumqi, Xinjiang Uygur Autonomous Region, China.

**Keywords:** gene mutation, thyroid hormone receptor, thyroid hormone resistance

## Abstract

**Rationale:**

Thyroid hormone resistance syndrome (THRS) is an inherited condition characterized by reduced responsiveness of target tissues to thyroid hormone. Due to their nonspecific symptomatic manifestations, these patients can be misdiagnosed. This study reports a pedigree with THRS caused by a mutation in the thyroid hormone receptor β (THRβ) gene.

**Patient concern:**

The proband, a 36-year-old woman at 19+4 weeks of gestation, was referred to our hospital because of abnormal thyroid function results. She was diagnosed with hyperthyroidism in October 2015, and had been treated with methimazole until her pregnancy.

**Diagnosis:**

The proband and 2 of her children were diagnosed with THRS based on genetic analysis. Sequence analysis of the THRβ gene showed a heterozygous mutation C>A located at exon 10. The mutation results in a change in proline for threonine at amino acid position 453, P453T.

**Interventions:**

No treatment will fully and specifically correct the defect. All 3 patients were in normal metabolic status, and thus treatment was not required.

**Outcomes:**

During a 2-year follow-up period, none of them had any complaints. The 20-year-old son (167 cm in height) and the 18-year-old daughter (150 cm in height) both had low academic performance.

**Lessons:**

Elevated serum thyroid hormone (TH) levels associated with nonsuppressed thyroid-stimulating hormone (TSH) levels usually leads to the diagnosis of THRS. Genetic analysis provides a short cut to diagnosis and the treatment should be based on the patient's clinical manifestations.

## 1 Introduction

The thyroid hormone resistance syndrome (THRS), an inherited condition characterized by persistently elevated levels of free thyroxine (FT_4_) and free triiodothyronine (FT_3_) levels, while normal or mildly elevated thyroid-stimulating hormone (TSH) levels, was first reported in 1967.^[1]^ Mutations in the thyroid hormone receptor β (THRβ) gene are responsible for 80% to 90% of THRS cases.^[2]^ Here, we describe a case of THRS that was suspected based on thyroid function findings. The diagnosis was confirmed by sequence analysis of the THRβ gene, which showed a heterozygous missense mutation C>A located at exon 10.

## 2 Case presentation

### 2.1 Proband

A 36-year-old woman at 19+4 weeks of gestation was referred to Xinjiang Medical University affiliated First Hospital for abnormal thyroid function in August 2017. She was tested for thyroid function a week ago because of excessive sweating. There was no history of fatiguability, weakness, proptosis or diplopia, palpitations, unusual bowel habits, or recent changes in weight. Upon physical examination: height 152 cm, body weight 60 kg, normal development, moderate skin temperature and elasticity, negative eye symptoms; a diffuse goiter with several small nodules, no tenderness, tremor or vascular murmur; heart rate 88 beats/min, no arrhythmia; no hands shaking or edema in lower extremities. Laboratory findings: thyroid function is listed in Table 1. Thyroid ultrasound showed a normal thyroid gland with some nonspecific nodules. The patient refused to undergo magnetic resonance imaging of the pituitary. The results of complete blood count, routine urinalysis, blood glucose, and renal function were all within normal ranges. She had no family history of thyroid disease. Past history showed that the patient had been diagnosed with hyperthyroidism for 2 years. Two years ago, she visited the hospital for lower abdominal pain and was diagnosed with hyperthyroidism after related inspections. Previous medical records showed abnormal thyroid function indicated by increased serum FT_3_ and FT_4_ levels, whereas TSH levels remained normal or mild elevated (Table 1). The radioiodine^131^ uptake test was completed in October 2015, which was 4.1% at 3 hours and 20.5% at 24 hours. A thyroid ultrasound showed a normal thyroid gland with several nonspecific nodules. She was treated with methimazole orally once per day for 10 mg. The proband visited the hospital several times and the methimazole dosage had been repeatedly adjusted (10–30 mg/d). However, thyroid function was not restored and medication was discontinued by herself because of pregnancy. During hospitalization, she was diagnosed with THRS based on genetic analysis and no medication was given.

**Table 1:**
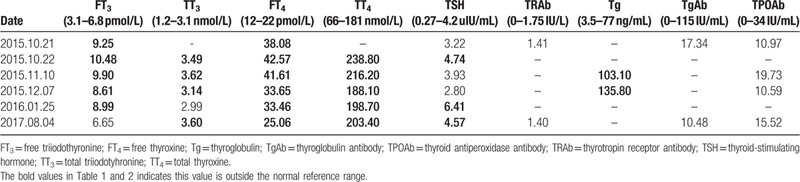
The proband's thyroid function panel from 2015 to 2017.

### 2.2 Kindreds

**Figure 1:**
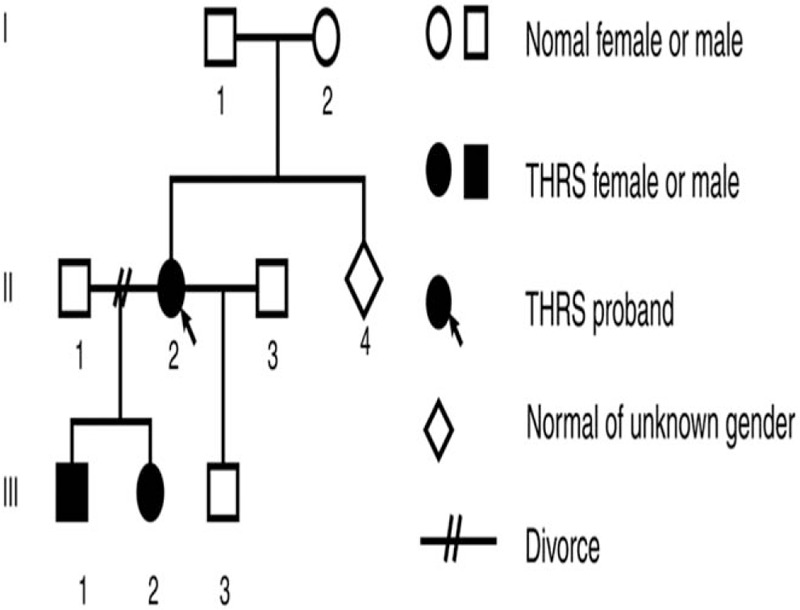
The pedigree of thyroid hormone resistance syndrome.

Three generations of the pedigree are presented in Fig. 1. The family had no history of consanguineous marriage. Neither the proband's parents nor her sibling had any history or symptoms of thyroid dysfunction, but their thyroid function and genotype were not available. Her eldest son (III:1) and daughter (III:2), aged 18 and 16 years, respectively, did not have any symptoms of hyperthyroidism or hypothyroidism, but they were shorter than their peers. The eldest son had a height of 167 cm (approximately –1SD on standard growth charts) and the daughter had a height of 150 cm (approximately –2SD on standard growth charts), while both had low academic performance. Their thyroid functions suggested that serum TT_3_, FT_3_, TT_4_, and FT_4_ levels were increased, while TSH levels were mildly elevated. The proband's youngest son was 49 cm tall weighted 3.6 kg of an uneventful 40-week gestation and normal vaginal delivery. His thyroid function was normal. No abnormal signs on physical examination were found in these 3 children. The thyroid function results are shown in Table 2. During a 2-year follow-up period, 3 children had no complaints and their physical exams were normal.

**Table 2:**
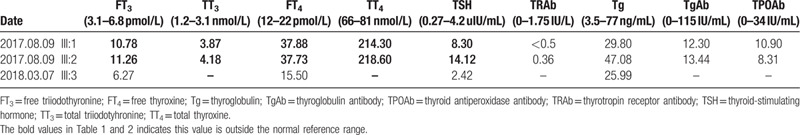
Thyroid hormone levels of the proband's kindred.

## 3 Methods

### 3.1 Data collection

Clinical materials and peripheral blood samples of the proband and her children were collected. Thyroid function parameters were detected by chemiluminescence immunoassay (Roche cobas6000). This study was ethically prepared and approved by the ethics committee of Xinjiang Medical University affiliated First Hospital, and the informed consent was obtained from all subjects for the publication.

### 3.2 Genetic detection

The peripheral venous blood (10 mL) was obtained from the proband and her children, and was sent to Ruijin Hospital, Shanghai Jiaotong University School of Medicine for gene sequencing. PCR-DNA sequencing was used to examine all 10 exons of the THRβ gene. The primers of 10 exons and adjacent intronic were designed according to the THRβ gene sequence provided by NCBI (NM_000461.4). Primer synthesis, PCR reaction, and sequencing were completed by the Shanghai Institute of Endocrine and Metabolic Diseases. The sequencing results were compared with the NM_000461.4 sequence to identify the mutation sites.

## 4 Results

PCR amplification of the THRβ gene of the proband followed by gene sequencing showed a heterozygous missense mutation c1357C>A located at exon 10. The mutation caused a change in thyroid hormone receptor β protein amino acid 453 proline to threonine, p.Pro453Thr. Sequencing exon 10 of her eldest son and daughter found that they both had p. Pro453Thr heterozygous pathogenic variation in THRβ gene. The youngest son did not undergo gene sequencing because of the presence of a chemically euthyroid.

## 5 Discussion

THRS, characterized by decreased thyroid hormone sensitivity in different degrees to the target tissues, is a rare genetic disease with an incidence of approximately 1/40,000. Its characteristic manifestations include goiter, physical deformity, learning disability, inattention, mental abnormality, and mental retardation. THRS is a rare cause of thyroid dysfunction and is prone to misdiagnosis and missed clinical diagnosis. Therefore, it is necessary to summarize and analyze this rare case.

Thyroid hormone receptors are encoded by 2 genes (THRα and THRβ), and THRS is usually caused by mutations in THRβ.^[3,4]^ The majority of the mutations are located in 3 clusters enriched with CpG dinucleotide hot spots in the carboxy terminus of THRβ.^[5]^ The THRβ gene contains 10 exons and encodes a total of 461 amino acids. Among them, exons 7 to 10 encode amino acids 178 to 461, which constitute the carboxy-terminal ligand-binding domain and part of the hinge region. Almost all THRβ mutations discovered so far are concentrated in the 3 “hot spots” between exons 7 and 10 (234–282, 310–353, 429–461), and only A229T, R243W, and R243Q in the mutation site are located in exon 7, most located in exons 9, 10.^[6,7]^ The 5 most frequently reported mutation sites are: R338W, A317T, R438H, R243Q, and P453T.^[8]^ In recent years, the point mutations reported in China include A297T, R383H, and M442T.^[9–11]^ The THRβ gene mutation found in our case is located in exon 10 at P453T, which is one of the hot spots reported abroad. It has only been reported in another Chinese girl with the same mutation.^[12]^ At present, >160 mutations have been identified.^[13]^ Previous studies have confirmed that there is no clear correspondence between THRS genotypes and phenotypes. Different families, patients in the same family, and different ages of onset can have different clinical manifestations.^[14]^ Formerly, THRS was categorized into generalized, isolated pituitary, and peripheral tissues. Based on symptoms and signs, this classification has not a logical basis now, because the former 2 are encountered in individuals with the same mutation and the latter represents the development of tolerance to the ingestion of excess thyroid hormone.^[15]^

The variable phenotype of THRS has multiple mechanisms. The clinical manifestations of THRS are highly heterogeneous, including hyperthyroidism, hypothyroidism, and nontoxic goiter.^[16]^ Goiter is the most common sign of THRS, and it is often the primary cause of medical attention. Other common symptoms include hyperactive behavior, learning disabilities, developmental delay, and sinus tachycardia. The severity of the disease is also uneven, ranging from asymptomatic to extremely severe symptoms, especially in childhood. Therefore, attention should be paid to THRS screening in children with the above clinical manifestations. At the same time, most THRS patients have familial heritability, so attention should also be paid to the screening of patients with a positive family history. Genetic diagnosis is currently the most reliable method for diagnosing patients with THRS. However, the gene defect in 15% of subjects with THRS remains unknown. The diagnosis cannot be ruled out for patients with suspected THRS who do not find mutations in genetic testing, and further tests, such as the TRH stimulation test, somatostation suppression test, and T_3_ test, must be combined to confirm the diagnosis.^[17]^

THRS is a hereditary disease and there is currently no cure for it. Fortunately, in most subjects with THRS, the partial tissue resistance to TH is adequately compensated for by an increase in the endogenous supply of TH and thus treatment is not required. Most importantly, it is not to intervene with the sole purpose of normalizing the TH levels.^[18]^ The patient's symptoms and clinical picture should be concentrated instead of normalizing their thyroid function. The clinician should consider the patient's age, symptoms, and response to previous treatments. Sinus tachycardia is very common and can be controlled with β-blockers. Regarding goiter, super physiological doses of L-triiodothyronine given every other day aiming to suppress TSH can reduce the size of the gland and the effective level of TSH suppression is assessed by the suppression of serum Tg.^[19]^ THRS patients with hypothyroidism should be treated with TH starting with a small dose and gradually increasing the dose. The effective dose varies from person to person. Children with growth and developmental disorders, mental retardation, and delayed bone healing should be diagnosed early and treated with a high dose of TH to maintain normal intelligence and growth. Factors such as linear growth, bone maturation, and mental development must be considered when adjusting doses of drugs.^[5]^

In conclusion, we have reported a pedigree of THRS with heterozygous P453T mutation in the THRβ gene. The diagnosis of this syndrome is based on genetic analysis. The treatment of THRS should be based on its clinical manifestations, not the need to normalize thyroid hormone levels. Our findings provide insight into the diagnosis and treatment of THRS.

## Author contributions

**Conceptualization:** Ming-Chen Zhang.

**Data curation:** Ming-Chen Zhang, Ayiguli Yusufu.

**Funding acquisition:** Ming-Chen Zhang.

**Investigation:** Ayiguli Yusufu.

**Methodology:** Ming-Chen Zhang.

**Resources:** Ming-Chen Zhang.

**Software:** Ayiguli Yusufu, Wen-Jing Chen.

**Writing – original draft:** Ayiguli Yusufu.

**Writing – review & editing:** Ming-Chen Zhang.
